# Sports related concussion: an emerging era in digital sports technology

**DOI:** 10.1038/s41746-021-00538-w

**Published:** 2021-12-02

**Authors:** Dylan Powell, Sam Stuart, Alan Godfrey

**Affiliations:** 1 Department of Computer and Information Sciences, Faculty of Engineering and Environment, Northumbria University, Newcastle upon Tyne, NE1 8ST UK; 2 Department of Sports, Exercise and Rehabilitation, Northumbria University, Newcastle upon Tyne, UK

**Keywords:** Physical examination

## Abstract

Sports-related concussion (SRC) is defined as a mild traumatic brain injury (mTBI) leading to complex impairment(s) in neurological function with many seemingly hidden or difficult to measure impairments that can deteriorate rapidly without any prior indication. Growing numbers of SRCs in professional and amateur contact sports have prompted closer dialog regarding player safety and welfare. Greater emphasis on awareness and education has improved SRC management, but also highlighted the difficulties of diagnosing SRC in a timely manner, particularly during matches or immediately after competition. Therefore, challenges exist in off-field assessment and return to play (RTP) protocols, with current traditional (subjective) approaches largely based on infrequent snapshot assessments. Low-cost digital technologies may provide more objective, integrated and personalized SRC assessment to better inform RTP protocols whilst also enhancing the efficiency and precision of healthcare assessment. To fully realize the potential of digital technologies in the diagnosis and management of SRC will require a significant paradigm shift in clinical practice and mindset. Here, we provide insights into SRC clinical assessment methods and the translational utility of digital approaches, with a focus on off-field digital techniques to detect key SRC metrics/biomarkers. We also provide insights and recommendations to the common benefits and challenges facing digital approaches as they aim to transition from novel technologies to an efficient, valid, reliable, and integrated clinical assessment tool for SRC. Finally, we highlight future opportunities that digital approaches have in SRC assessment and management including digital twinning and the “digital athlete”.

## Introduction

Direct impact(s) to the head or neck during contact sport are major contributors to individuals sustaining Sports-Related Concussion, SRC^1^. The incidence of SRC has grown in many contact sports. For example, in rugby union, the incidence can be as high as one concussion per game^2,3^. Accordingly, SRC presents notable health risks to those participating in contact sports where the intensity of e.g., high impact collisions are commonplace with considerable challenges in diagnosis and monitoring to inform return to play/participation (RTP)^3–6^.

Timely identification of SRC is of critical importance to SRC management, to avoid adverse neurological implications^4^. Appropriate SRC management ensures participants do not RTP prematurely as this can lead to a secondary brain injury^1,2,5^. The second impact can have serious consequences including increased intracranial pressure and in extreme cases, death^6^. Hence, diagnosing SRC through timely and accurate assessment is of crucial importance to minimize short-term health risks. This is reinforced by evidence highlighting the potential long-term impacts of inappropriate SRC management on brain health and chronic traumatic encephalopathy (CTE)^7,8^. Long-term neurological deficits associated with head trauma have increased public health concerns (across many sports), driving demand for evidence-based monitoring and treatment^9^.

Immediate and accurate (on-field) recognition and management of SRC remain difficult. This includes professional teams/sports that often possess sufficient medical staff to monitor for suspicious mechanisms of injury which may lead to a SRC^10,11^. Thus, accurate recognition of SRC is particularly challenging in environments with limited medical support such as amateur teams/sports, where there may be one coach or first aider only. In rugby union environments with reduced medical provision, the conservative approach of “Recognize & Remove and if in doubt, sit them out” is adopted^12^. That involves permanently removing players identified as being involved in possible head injury-related events (e.g., contact with head or neck) or if they display signs and symptoms associated with SRC there is no return to sporting activity until a medical assessment is performed. That aims to reduce occurrences of missed or misdiagnosed SRC in low-resource/amateur environments.

SRC presentation is heterogeneous with a wide variety of signs and symptoms, some of which are subtle and easily missed or may only become apparent in the following hours and days after injury^13^. Therefore, challenges remain in the subsequent (off-field) assessment and RTP protocols following SRC. This is confounded by traditional approaches used to diagnose and monitor SRC often occurring during infrequent snapshot assessments. The most widely used approach in SRC assessment is the Sports Concussion Assessment Tool (SCAT) which tests aspects of cognition, balance, and vision via a paper-based questionnaire administered by a health professional^1,14^. The manual but subjective nature of tests like SCAT, means formal SRC diagnosis, rehabilitation, and RTP is based solely on clinical judgment with information gathered from self-reported assessment techniques^15^. This is problematic as research shows SRC is a dynamic and complex pathological process with difficult to measure impairments that can change or deteriorate rapidly without any prior indication^16^. This presents challenges for the safety, rehabilitation, and RTP.

The Concussion Consensus Statement^13^ reinforces a need for more objective approaches through robust development and provision of diagnostic and prognostic biomarkers to better assess the presence/severity and recovery of SRC, respectively. Digital imaging technologies such as functional MRI (fmRI) or pET scanners are (reference standards) already used to assess the severity of damage such as skull fractures and bleeding on the brain in more severe traumatic injuries. However, their effectiveness and practicality when used in isolation for SRC diagnosis is yet be proven, with only a minority of mTBI such as SRC displaying distinguishable structural changes immediately post concussion^17^. In addition, not all players suspected of SRC require hospital assessment and of those attending Accident and Emergency (A&E) Departments, only those presenting the most severe signs and symptoms will be sent for imaging^18–20^. Thus, those reference technologies are not typically deployed for routine SRC assessment.

Recently, lower-cost (digital) technologies have been developed to measure and monitor outcomes for more informed assessments^21^. Such approaches could provide scalable robust data for more informed and integrated SRC diagnosis to better inform RTP, enhancing the efficiency and precision of healthcare assessment^22,23^. In this narrative review, we examine SRC clinical assessment methods in four key areas (cognitive, visual, motor, symptom), providing insights into the translational utility of readily attainable digital methods. We examine common benefits and challenges facing those digital approaches as they aim to transition from novel technologies to efficient, valid, reliable, and integrated clinical tools for SRC. We highlight future opportunities that attainable digital tools can have in SRC diagnosis and monitoring with a systems-science-based management approach including digital twinning and the “digital athlete”. We provide recommendations on how this field should develop.

## Sports-related concussion assessment

The rise in SRC cases presented at A&E/ED has prompted closer discussion about improved assessment and management, including calls for the development of national guidelines^18^. Mistry et al.^20^ highlight the main objective of SRC assessment in A&E is to triage the player/patient, identifying any readily obvious brain injury symptoms/signs that require e.g., surgical intervention. That approach, although it may improve efficiency, omits thorough assessment of many other subtle SRC impairments such as cognitive, motor/functional (e.g., balance, gait), and visual deficits^24^. Thus, current SRC assessments are often binary snapshots, ignoring the interconnected nature and heterogeneity among individuals. Most post-discharge management involves information for the player regarding red flag signs/symptoms and/or provision of head injury information leaflets. Furthermore, outside of professional environments, there is often no physician assessment or follow-up until returning to full contact training^25^.

As such current SRC management and rehabilitation protocols rely on self-reported measures/symptoms to determine readiness to play. Therefore, SRC recovery times and prognosis is highly variable and varies dramatically across different age groups and gender. Indeed some individuals can take significantly longer than expected to RTP (3-4 weeks) and experience chronic symptoms even after returning to play^26,27^ As such reliance on subjective non-specific measures such as symptoms make it extremely difficult to confidently know when it is safe for players to RTP. This highlights the need for valid, objective tools to aid diagnosis, monitoring, and RTP in SRC^20^.

### Cognitive: routine clinical approaches

Comprehensive assessment of cognitive function outside of sport typically includes detailed interviews, exploring the history of a patient’s health, education, and social background. In contrast, SRC focuses on more specific areas of cognitive functioning only such as short-term memory, working memory and executive-level function^28^. Pen-and-paper tests include the short-blessed test, digit span (forward and/or reverse) and the Standardised Assessment of Concussion (SAC), now incorporated into the fifth version of the SCAT (SCAT5). Despite widespread clinical use, these tests carry considerable challenges including manual score calculation hindering automated or immediate comparison of scores across different individuals and time points^29^. Fortunately, progression to using digital neurocognitive testing has overcome some of these limitations.

### Cognitive: digital approaches, computerized programs

The introduction of digital-based cognitive assessments offers a number of advantages over pen-and- paper methods including objective cognitive metrics (e.g., reaction time calculation), randomization of test trials with automation of data collection and analysis^30^. Immediate Post-Concussion Assessment and Cognitive Test (ImPACT) is an example of a scalable computerized neurocognitive tool that assesses verbal memory, reaction time, visual-motor speed, and visual memory^31,32^. ImPACT tests are complemented with the integration of demographic data and a post-concussion symptom scale for players and staff. Research shows ImPACT is sensitive post concussion in the acute phase (within the first few days) with measurable differences in verbal memory, visual memory, and slower reaction times^33,34^. However, there is mixed evidence for neurocognitive testing in subacute and chronic concussion^3,25,35^. Indeed, the international consensus statement of concussion states that “tests should not be seen as the sole basis for the management of decisions”^4^.

Despite the value of digital neurocognitive testing in acute SRC cognitive testing, challenges remain for pragmatic deployment in low-resource environments. The high cost of initial software licenses or fixed yearly subscriptions can be prohibitive to amateur sports teams with limited budgets. Often these commercially orientated companies rarely permit independent validation of their technologies or algorithms used to interpret raw data or outputs. This lack of an open-source or transparent approach makes it very difficult for governing bodies to make evidence-based decisions about which test or technology to endorse/promote. Other pragmatic limitations on a single approach are the reliance on baseline data, where it isn’t always feasible to gather pre-injury data due to e.g., players moving between clubs/teams. Without baseline information, it is difficult to ascertain if an athlete’s post-concussion neurocognitive scores are the result of a concussion or individual variability. Consequently, no single cognitive test/technology has proven capable for standalone use. This has placed greater responsibility on clinicians to have prior experience and use clinical judgment when managing SRC^13,33^, which may partly explain the reluctance to adopt technology in SRC assessment.

### Visual: current approaches

Normal vision correlates with healthy cerebral activity and brain function^36^. SRC can cause impairments in visual and oculomotor speed, with research showing oculomotor dysfunction present in up to 90% of SRC cases^37^. Traditional subjective visual assessment includes eye-tracking tests e.g., Visual Occulomotor Assessment (VOMS), which assesses impairments via self-report. This test includes a baseline measurement where players verbally rate changes in headache, dizziness, and nausea symptoms compared with their immediate baseline state on a scale from 0 (none) to 10 (severe) to determine if each test provokes symptoms^38^. Other visual tests include the King-Devick (K-D), which is an indirect measurement of rapid eye movements, language function, and attention. The K-D test has demonstrated moderate sensitivity (60%) but poor specificity (39%) in identifying players diagnosed with concussion^35^. It is also unclear how training and learning effects can influence participant scores, and to date, there is an absence in clinically significant change scores/data. Indeed a recent paper outlined that current eye-tracking tests (such as K-D) were no better than traditional off-field screening alternatives^35^. This is confounded by deficiencies and heterogeneity in current cognitive testing protocols and environments, making comparisons between studies and decisions on the choice of test difficult^34,39^. As such the paper advised that current tests should not be routinely incorporated in SRC assessment.

In addition to suboptimal sensitivity, current tests rely heavily on baseline data collection which are not feasible to implement in low-resource environments, where there is often insufficient staff/funding to perform baseline screening. Hence there is significant demand for more sensitive, objective, and scalable solutions for visual assessment in the form of wearable digital eye trackers and/or mobile technologies.

### Visual: wearable digital eye trackers

Non-invasive digital technologies such as eye trackers can objectively monitor eye movements during laboratory tasks, assessing visual and cognitive processing^36,40^ in a variety of research paradigms ranging from neuroscience to social science^41^. Despite this rapid rise in the availability of technologies, there are several barriers to clinical deployment. Stuart et al.^42^ outlined current state of the art and challenges in mTBI visual assessment, finding most studies do not adequately address or report the validity or reliability of eye trackers, making a comparison or clinical interpretation difficult. To translate these technologies into clinical application there is a need for more routine validation, standardization in testing paradigms, and transparency on their use, including algorithms and data analysis methods^43,44^.

For the few studies with adequately reported information, Khalife et al. highlight the benefits of investigating rapid, reliable eye-movement impairment in SRC assessment with the Tobii eye-tracker^45^. The latter shines a light onto the eye causing a reflection, a high-resolution camera then captures an image of the eye with reflections which is then used to calculate gaze direction. Research has found the accuracy of the Tobii EyeX to offer sufficient accuracy and precision in gaze direction^46^. This is consistent with research testing other technologies such as the Eye-Sync which offer good-excellent levels of sensitivity (88%) and specificity (87%) in smooth pursuit assessment^47,48^. Overall digital technologies offer high-resolution quantitative data and value over traditional approaches such as the VOMS. Despite promising results of accuracy in academic research, there has yet to be clinical research investigating thresholds or measures that can be applied to a meaningful change that can be widely used for SRC assessment.

### Motor: balance and gait under direct observation

Balance and gait/walking impairments are associated with neurological conditions, including concussion, and therefore forms a key component of clinical assessment^1,49–51^. The Balance Error Scoring System (BESS) test is a balance and postural stability assessment that is widely used for examining impairments by asking participants to specific adopt stances aimed to challenge their motor and vestibular system^52–54^. However, the BESS is assessed subjectively, through manually recording errors (e.g., if the participant removes a hand from their waist during a single leg stance) and timed using a stopwatch. As a consequence, the BESS sensitivity is greatly influenced by assessor experience and research suggests only sensitive in the acute phase (within first 2 days of injury)^55^. These inherent limitations of subjective assessment make it difficult to apply in sporting environments, where there is demand for precise and sensitive clinical measurements. As outlined by Johnson et al., traditional balance assessments “are subjective in nature, do not adequately challenge high functioning athletes and may not be capable of detecting subtle balance disturbances following a concussive event”^56^. This raises questions surrounding the accuracy in diagnosis, RTP protocol and crucially, paradigms by which SRC is assessed. Indeed gait and postural deficits may in fact be impaired for long periods beyond, typical timeframes of recovery^57,58^. Therefore, traditional assessment of motor function carries significant limitations yet remain extremely prevalent across the clinical practice. Opportunities for improvement may be afforded by adopting digital approaches such as inertial sensor-based wearables discussed in the next section.

### Motor: inertial sensor-based wearables

The development of wearables equipped with inertial sensors (accelerometers and gyroscopes) has facilitated pragmatic instrumented testing of traditional approaches such as the Timed-Up-and-Go (iTUG) and BESS^50,59,60^. These studies do show attempts to instrument traditional tests and provide objective digital SRC biomarkers from a single wearable sensor. Recently, Celik et al.^61^ adopted a multi-wearable approach towards a comprehensive instrumentation of SCAT5. By using eight inertial wearables (wrists, legs, lower back) to segment-specific components (e.g., tandem walk and static balance) a wealth of spatial and temporal data associated with each SCAT5 component with excellent/millisecond resolution. Moreover, the study showed how wearables can automatically and more accurately calculate, recognize balance and gait errors during tasks compared to clinical observation, also highlighted by Johnston et al.^58,62^. Beyond instrumentation of traditional assessment, research with inertial wearables shows SRC and mTBI impacts balance, gait, and turning^55,63–66^, including under longitudinal assessment^55^.

Despite laboratory research showing motor impairments can be strongly associated with SRC, it is not yet known exactly what clinical gait or turning assessment techniques are sensitive for SRC. Therefore, barriers remain in clinical validation and how to translate some novel inertial measures (e.g., frequency-based data) into clinical endpoints or biomarkers. Indeed, the episodic nature of current laboratory assessments may be supplemented beyond the clinic/hospital during real-world/free-living remote assessment^67,68^. However, a necessary precursor to longitudinal free-living remote balance and gait assessment is verified and validated digital SRC biomarkers enabling trust and better understanding by clinicians and patients^43^.

### Symptom: current approaches

Despite rapid and extensive development in the availability of different tests to assess SRC, the symptom checklist and severity indices are retained as the cornerstone for most decisions around readiness to return to play. This includes the Post-Concussion Symptom Scale (PCSS), which assesses a variety of symptoms (0-6 of increasing severity) to give an overall score and has been adapted and abridged into 5th edition of the SCAT (SCAT5)^54,69^.

Although common due to their ease of use, studies have examined the sensitivity of symptom scales in SRC and found suboptimal sensitivity and specificity^54,55,69^. Moreover, the severity of symptoms/signs reported by players following a SRC varies significantly (immediate or delayed onset) which can hinder confidence for clinicians and players/patients when assessing readiness to return to play^54,70^. In addition, some studies report that players can manipulate self-reported baseline symptom scores, allowing them to mitigate any poorer performance of scores post concussion^71^. For example, only 17% of athletes self-reported symptoms of SRC, although nearly half of this cohort (48%) sustained a head injury and associated signs of SRC^71^. Relying on self-reported data may be particularly challenging in competitive environments where there is societal or financial gain in staying injury-free.

Alongside the challenges of subjectivity in self-reported symptomatology, there are significant practical and logistical barriers. Current pen-and-paper-based SRC assessment methods can take 10–15 min/player which is not always achievable in environments with only one medical practitioner to complete player assessment (e.g., at an amateur level)^72^. Therefore, challenges remain in providing approaches to more efficiently document SRC injury characteristics across both low- and high-resource environments. The growth in usage and availability of smartphones and affiliated commercial digital technologies means players and clinicians already have widespread access and familiarity with use. A move toward mobile digital applications may serve to overcome some limitations of symptom assessment, data storage, and analytics compared to self-reported pen-and-paper methods.

### Symptom: digitally recorded symptoms

Several smartphone/mobile digital applications/apps are available to track injuries and monitor SRC recovery through symptom reporting. Apps include CSX (used by e.g., World Rugby) and the Cleveland Clinic Concussion Application (C3) which records data on reaction time, memory, vision and information processing^73^. To the author’s knowledge, CSX has yet to be fully deployed into amateur sports. However, C3 has been used to collect some concussion data in college and professional rugby^72^. Linder et al. found that use of an Electronic Injury Reporting (IR) app provides a useful digital platform for injury-related demographic analysis^72^. App advantages include the capacity for players to complete (in their own time) symptom recording as frequently as required with more regularity and consistency in the absence of clinicians.

The current reliance on traditional non-digital approaches such as the SCAT5 and lack of robust databases means the progression and recovery of SRC symptoms is unclear^1,35^. Moving toward digital symptom recording may allow greater understanding and co-investigation with other SRC impairments.

## Towards daily use of digital approaches

**Fig. 1:**
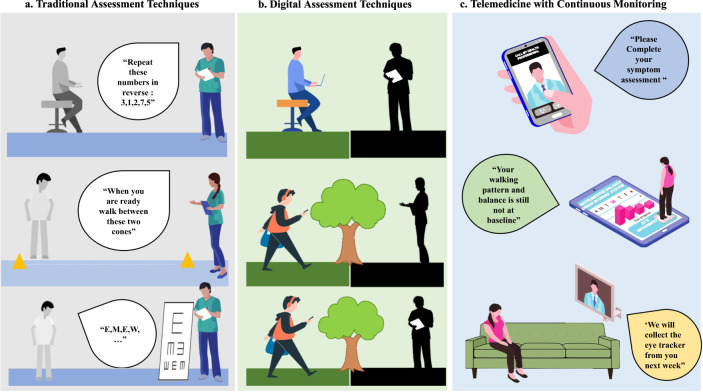
Contrasting traditional to digital approaches. **a** Traditional approaches to assessment rely on subjective/pen-and-paper tests which may be administered by different healthcare professionals, introducing scoring bias. **b** Use of digital technologies empower the player/patient to perform test during activities of daily living where e.g., wearables could provide continuous monitoring of motoric tasks like gait/walking (i.e., more emphasis on the player). **c** Digital technologies would enable remote monitoring for longitudinal assessment in habitual settings. This figure has been designed using resources from Freepik.com author, Makrovector: people vector (www.freepik.com/vectors/people), Computer vector (www.freepik.com/vectors/computer), School vector (www.freepik.com/vectors/school).

Digital approaches and technologies could provide objective information, generating useful and reliable data for improved data presentation, analysis, and insights for SRC management. From this narrative review, Fig. 1 presents a hypothetical scenario contrasting traditional (1a) to digital (1b) approaches for cognitive, motor, and visual assessment. Figure 1a alludes to current limitations e.g., different clinicians performing assessments in highly controlled and supervised environments. Figure 1b demonstrates how digital-based assessments could facilitate integration from multimodal sources (technologies, inc. wearables). This multimodal digital approach could capture e.g., behavioral trends continuously and remotely in habitual environments without the need for a clinician to be present. Fig. 1c. By adopting complimentary digital approaches, more objective comparisons or insights could be made across a range of SRC impairments (cognitive, visual, symptom, and motor).

## Discussion

Implementing digital approaches in sports medicine and SRC care could transform how player data is captured, analyzed, and communicated. Current SRC approaches are restricted by the reliance of subjective self-reported assessment under direct observation of a clinician. Thus, outcomes are often reliant on a player informing the clinician and the clinician’s clinical judgment or interpretation. Objective approaches in SRC are often confined to bespoke or professional environments, limiting deployment and accessibility to amateur or adolescent players. In addition, there is considerable focus on traditional in-person assessment at episodic “snapshot” assessments with little to no remote/habitual data collected on those who sustain a SRC during contact sports. The addition of digital or remote assessment approaches such as wearables may augment, and supplement data gathered in traditional assessment visits under the supervision of healthcare professionals.

Presently, digital cognitive testing only offers a snapshot assessment. Yet, testing could be better utilized through constant remote evaluation via apps. This would mitigate the need for clinicians to be present and would allow a higher frequency of testing within the players routine environment^74^. Although testing in the latter would be conducted in less controlled conditions, there is considerable value in conducting testing in remote, real-world/free-living as they would be within habitual conditions^21,75,76^.

The use of current eye-tracking approaches in SRC is not currently supported. However, there are opportunities for use of digital eye-tracking outside of the clinic or in static situations. As these methods do not require active participation of the wearer, they overcome many potential issues of adherence allowing participants to wear these technologies as part of their daily life^21^. However, capturing reliable data on impaired eye movement outside of controlled/laboratory conditions generates many complications. Thus, challenges remain in the refinement and optimization of eye-tracking as a future SRC diagnostic tool. These include minimum and maximum testing times, choice of eye-tracking tests, lack of standardized protocols to detect SRC eye-movement impairments as well as the complexity of analyzing big data. Overcoming these challenges will require the development and refinement of protocols and data processing methods/algorithms.

Use of (inertial-based) wearables within mTBI has shown considerable promise for measuring balance and gait impairments^53,63,77–79^. Yet the true utility of inertial technologies may be their use beyond the clinic with the provision of habitual balance and gait data^67^. Such wearables should become more accepted and the standard for gathering continuous, high-resolution free-living data due to their discrete attachment and low wearer burden. Technical validation of inertial wearables has led to the development of a conceptual gait model^80^, providing a framework for clinicians to better utilize gait data to make more informed clinical decisions. For example, a similar modeling approach^78^ has been applied in chronic (non-sporting) mTBI providing enhanced gait analysis, which could be a means to assess response to interventions and better understand underlying impairments. Future research should apply and evaluate conceptual models in acute mTBI and SRC from free-living gait data to provide better insight to habitual player recovery, better informing RTP.

Symptoms post SRC are thought to be closely linked to improvements in physiological recovery and should therefore remain a cornerstone of assessment^81,82^. However, digital monitoring may not easily lend itself within free-living due to the requirement for players' attention. Yet by collecting longitudinal (habitual) symptom data, a deeper understanding of the rate of progression of symptoms could be determined, supporting the transition and deployment of other digital approaches. Clinicians could deploy apps to measure symptoms beyond snapshot testing points but would need to account for testing conditions. Adoption of mobile technologies to support symptom documentation would allow integration with other digital approaches, providing holistic systems-based approaches to SRC management. If used routinely, such approaches may have capacity to provide alert systems to healthcare professionals for missed SRC or injuries within squads, which could standardize and systematize injury severity through evaluation of red flags via structured and personalized assessment.

### Towards the digital athlete

Measuring and monitoring a single impairment is unlikely to reveal meaningful new insights into SRC. There is a need for a multidimensional/multimodal approach with digital diagnostic and prognostic models/frameworks to improve outcomes^83^. Therefore, step changes to understand, diagnose and manage SRC will require multi-scalar approaches which could be built around a systems-science framework to shift research into practice. Achieving this will require cross-disciplinary collaborations and the adoption of novel approaches with shared repositories to facilitate and intensify collaboration.

**Fig. 2:**
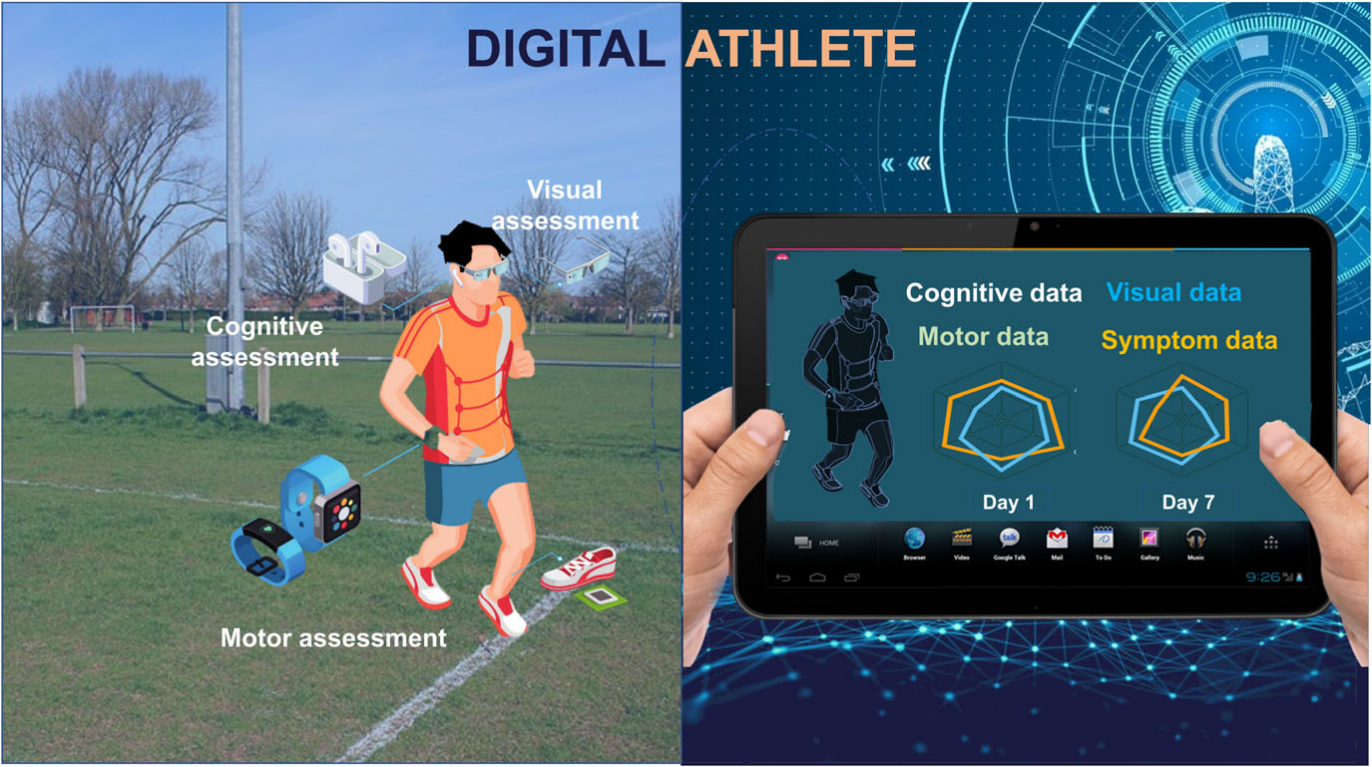
Digital technologies can enable the digital athlete. Capture visual outcomes through smart glasses, cognitive outcomes through voice-activated ear-pods, motor assessment with wearable movement monitors (inertial measurement units) on the wrist or embedded within equipment (e.g., shoe), while symptoms could be recorded through a personal smartphone (not shown), left. The digital representation (right) of the athlete enables high resolution and longitudinal data to be investigated, examining trends. (Parts of this figure utilizes photos from Unknown Authors, all licensed under CC BY-SA).

One emerging concept is digital twinning, a strategic technology made feasible through developments in the Internet of Things (IoT) and big data. It has been applied to complex systems and in medicine to provide a framework to create a virtual representation of players based on the integration of data from digital devices, omics, imaging, and electronic medical records^84^. A digital twin can represent a back-up/copy to a person’s physical state before an intervention, providing retrospective or real-time monitoring of a wide range of parameters^85^. The application of wearables to create a digital twin of baseline health information for a player participating in contact sport would provide objective data, providing opportunities for remote monitoring and evaluation^86^. This leads to the concept of the digital athlete (Fig. 2) where an open framework is proposed for the emerging areas of digital health^86^. The ubiquitous nature of IoT/digital technologies coupled with digital twinning offers the potential for a paradigm change to better understand mTBI and more effective detection, prediction, and assessment of SRC. However, digital twinning is not just about collecting data, it is also about creating the computing architecture allowing new insights to support decision making, synthesizing information, facilitating communication, and the development of shared hypotheses^87^. Incremental changes in the ability to gather data to generate biomarkers related to health would enable the creation of player-centric protocols and targeted treatments. Central to this development has been the recognition that wearables are now part of IoT systems, incorporating sensing with data analytics to create an integrated approach, providing insights into physiological status, health and performance^85,88^. Built on the concept of digital twinning, the digital athlete would enable better integration of data, simulation of scenarios, and predict outcomes more accurately for SRC assessment and monitoring.

### Future considerations: important next steps and recommendations

Digital approaches could have tangible objective improvements in SRC diagnosis and monitoring. However, there are notable application and deployment challenges pertaining to sports (individual versus team), funding, environments (professional and amateur), and education. This will demand different approaches to ensure correct adherence and implementation as well as robust data collection protocols to ensure adequate monitoring. Likewise, there are privacy (security), ethical (remote and/or continuous monitoring), and trust considerations (effectiveness of digital technologies to augment traditional approaches) when collecting SRC data. To better understand these demands, there is a need for independent and multidisciplinary research with diverse stakeholders (e.g., athlete/patient, clinician, technologist, and sport’s governing bodies) with transparency in findings and conclusions drawn. To support behavior change for routine digital adoption in SRC, there must be the development of multidisciplinary standardized frameworks and agreement in validated/reliable tools to ensure trustworthy technologies that are fit-for-purpose. Accordingly, high-level recommendations include:

- Routine engagement with sport-specific stakeholders on how digital tools could advance SRC diagnosis and monitoring.
- Development of open-source athlete digital monitoring approaches for routine integration of data streams.
- An expert, multidisciplinary consensus on the use of fit-for-purpose digital SRC tools within and/or across sports.
- Transparency of all digital tools in SRC assessment (i.e., no black-box development).

## Conclusions

The increasing incidence of SRC and challenges of current diagnosis approaches has illuminated the scale of the problem facing clinicians for routine diagnosis and monitoring. Although traditional and subjective approaches will remain a crucial component of SRC assessment, they are unable to reliably provide an evidence-based approach to the detection, monitoring, and management to inform RTP. Digital approaches have the potential to transform the way player/participant data can be objectively captured, processed, and analyzed, enhancing current healthcare practice in SRC. Informative digital biomarkers from habitual behaviors could be routinely captured providing reliable (big) data that can support the development of other novel SRC biomarkers. Adopting free-living assessment is feasible with some current wearables but future considerations should be given to integration with IoT platforms for a multi-model, remote and holistic player assessment. Digital-based approaches coupled with novel concepts/frameworks from other research domains (e.g., digital twinning) provide a persuasive and timely route to addressing ongoing limitations in SRC. Recommendations provided here could help modernize (digitize) SRC diagnosis and monitoring to protect athletes and their sport.
